# Polymorphisms of Cytochromes P450 and Glutathione S-Transferases Synergistically Modulate Risk for Parkinson’s Disease

**DOI:** 10.3389/fnagi.2022.888942

**Published:** 2022-04-29

**Authors:** Hui-Hui Fan, Bao-Qing Li, Ke-Yun Wu, Hai-Dan Yan, Meng-Jie Gu, Xing-Hao Yao, Hao-Jia Dong, Xiong Zhang, Jian-Hong Zhu

**Affiliations:** ^1^Department of Preventive Medicine, Institute of Nutrition and Diseases, Wenzhou Medical University, Wenzhou, China; ^2^Department of Neurology, Institute of Geriatric Neurology, The Second Affiliated Hospital and Yuying Children’s Hospital, Wenzhou Medical University, Wenzhou, China; ^3^Department of Clinical Laboratory, The Second Affiliated Hospital and Yuying Children’s Hospital, Wenzhou Medical University, Wenzhou, China

**Keywords:** metabolizing enzymes, Parkinsion’s disease, polymorphism, synergistic effect, genetic association

## Abstract

**Background:**

Environmental substances such as pesticides are well-known in link with Parkinson’s disease (PD) risk. Enzymes including cytochromes P450 (CYPs), esterases and glutathione S-transferases (GSTs) are responsible for the xenobiotic metabolism and may functionally compensate each other for subtypes in the same class. We hypothesize that the genetic effects of each class modulate PD risk stronger in a synergistic way than individually.

**Methods:**

We selected 14 polymorphic loci out of 13 genes which encode enzymes in the classes of CYP, esterase, and GST, and recruited a cohort of 1,026 PD and control subjects from eastern China. The genotypes were identified using improved multiplex ligation detection reaction and analyzed using multiple models.

**Results:**

A total of 13 polymorphisms remained after Hardy-Weinberg equilibrium analysis. None of the polymorphisms were independently associated with PD risk after Bonferroni correction either by logistic regression or genetic models. In contrast, interaction analyses detected increased resistance to PD risk in individuals carrying the rs12441817/CC (*CYP1A1*) and rs2070676/GG + GC (*CYP2E1*) genotypes (*P* = 0.002, OR = 0.393, 95% CI = 0.216–0.715), or carrying the *GSTM1*-present, *GSTT1*-null, rs156697/AG + GG (*GSTO2*) and rs1695/AA (*GSTP1*) genotypes (*P* = 0.003, OR = 0.348, 95% CI = 0.171–0.706). The synergistic effect of *GST*s on PD was primarily present in females (*P* = 0.003). No synergistic effect was observed within genotypes of esterases.

**Conclusion:**

We demonstrate a presence of synergistic but not individual impact on PD susceptibility in polymorphisms of *CYPs* and *GSTs*. The results indicate that the genetic interplay leads the way to PD development for xenobiotic metabolizing enzymes.

## Introduction

Parkinson’s disease (PD) is the second most common neurodegenerative disease, affecting 1.14 and 1.06% of people over 60 years old worldwide and in China, respectively (Pringsheim et al., 2014; Cui et al., 2020). Only a minority of PD are known with genetic causes and familial hereditary, such as mutations in *SNCA*, *LRRK2*, and *PARKIN*. In contrast, most PD cases are sporadic with unclear etiology. Complicated interactions between genetic susceptibilities and environmental exposures are considered to be responsible for the pathogenesis of sporadic PD (Kalia and Lang, 2015).

The chemical 1-methyl-4phenyl-1,2,3,6-tetrahydropyridine (MPTP) was discovered to induce parkinsonism in humans in the 1980s (Langston et al., 1984). MPTP was later found to be structurally similar to some known pesticides such as paraquat, raising the question whether pesticide exposures were associated with PD (Wirdefeldt et al., 2011). Multiple kinds and classes of pesticides have since been identified to link to PD development. For example, exposure to herbicide paraquat, insecticide rotenone or fungicide maneb leads to toxic effects similar to those of MPTP in animal models (Islam et al., 2021). Benomyl, another fungicide, inhibits aldehyde dehydrogenase activity, and results in accumulation of 3,4-dihydroxyphenylacetaldehyde (DOPAL), a dopamine metabolite highly toxic to dopaminergic neurons (Fitzmaurice et al., 2013). Organophosphate pesticides are also shown to be associated with increased risk of PD, probably due to the inhibition of acetylcholinesterase activity and the induction of oxidative stress (Sanchez-Santed et al., 2016). In addition, pesticide exposure exhibits the strongest association with PD among 11 environmental risk factors identified in a meta-analysis (Noyce et al., 2012).

Pesticides are metabolized *in vivo* by phase I enzymes such as cytochromes P450 (CYPs) and esterases into reactive metabolites. Phase II enzymes such as glutathione S-transferases (GSTs) then detoxify the reactive metabolites through catalytic conjugation with charged species such as reduced glutathione (Omiecinski et al., 2011). Genetic liabilities of these enzymes to diseases have been widely studied such as in context of diabetes, cancer and cardiovascular disorders (Gao et al., 2017; Elfaki et al., 2018). It has also been investigated whether the enzymes are genetically associated with PD. However, some of the results remain inconsistent and inconclusive, such as for *CYP2D6*, and *GSTM1* (Ahmadi et al., 2000; Chan et al., 2003; Elbaz et al., 2004; Perez-Pastene et al., 2007).

Since the same class of xenobiotic metabolizing enzymes may functionally compensate each other or overlap, we hypothesize that the genetic effects of each class modulate PD risk stronger in a synergistic way than individually. We selected 14 polymorphic loci out of 13 genes which encode metabolizing enzymes of three classes, GSTs, esterases, and CYPs (Supplementary Table 1). Amongst, *GSTM1* and *GSTT1* are two deletion polymorphisms, and the remaining are single nucleotide polymorphisms (SNPs). These polymorphisms are either functional in modulating protein expression or enzyme activity or are reportedly associated with PD risk (Supplementary Table 1). Their genetic effects on PD were herein investigated individually and synergistically in a large Chinese cohort.

## Materials and Methods

### Subjects

A total of 1026 Han Chinese subjects were recruited from eastern China, including 527 sporadic PD cases (270 males and 257 females) and 499 controls (270 males and 229 females). The median age of the cases and controls was 66 (interquartile range, 59–73) and 58 (interquartile range, 50–68), respectively. PD cases were diagnosed by two neurologists according to the UK Parkinson’s Disease Society Brain Bank Criteria (Hughes et al., 1992). Patients with a family history of PD, or with secondary and atypical parkinsonism were excluded. Control subjects were free of neurological disorders determined by medical history, physical and laboratory examinations. Informed written consents were obtained from all participants or their legal representatives. The study was approved by the Ethics Committee of the Second Affiliated Hospital and Yuying Children’s Hospital, Wenzhou Medical University.

### Genotyping

Genomic DNA was extracted from peripheral blood using TIANamp Genomic DNA kit (Tiangen, Beijing, China) according to the manufacturer’s instruction. The candidate polymorphisms were genotyped using the improved multiplex ligation detection reaction (iMLDR) at Genesky Biotechnologies (Shanghai, China). Specifically, the alleles of each polymorphism were distinguished by different fluorescent labels of allele specific oligonucleotide probe pairs. Different polymorphisms were further distinguished by different extended lengths at 3′ end. Four percent of the total samples were randomly selected and repeated as quality control, which showed consistent results.

### Statistical Analysis

All data were analyzed using the Statistical Package for Social Science program (version 23.0). The χ^2^-test was used to assess Hardy-Weinberg equilibrium in genotype distribution and the difference in gender. Following Kolmogorov-Smirnov test for normality, Mann-Whitney *U*-test was used to evaluate age difference. The differences in genotype and allele frequencies, as well as the interactions between polymorphisms were analyzed using logistic regression model with gender and age as covariates. Homozygous genotypes of low frequency less than 0.05 were with limited sample size and were then grouped into their respective heterozygous genotypes during the interaction analysis to increase the statistical power (Mazaheri et al., 2006; Oliveira-Paula et al., 2021). Genetic models were analyzed using SNPStats software at https://www.snpstats.net. A backward elimination method as reported previously was used to identify the highest-risk genotype combination for PD (Ionita-Laza et al., 2014). Significance was set at α = 0.05.

## Results

### Association Analyses of the Candidate Polymorphisms With Parkinson’s Disease Susceptibility

The PD cases and controls were comparable in gender (*P* > 0.05) but different in age (*P* < 0.05). Genotype distributions of the polymorphisms in controls met with Hardy-Weinberg equilibrium (*P* > 0.05), except for *CYP2D6-*rs1065852 (*P* = 0.002). This variant was then removed from later analyses. Differences of the remaining 13 variants in genotype and allele frequencies were analyzed between the PD cases and controls (Table 1). A difference (*P* < 0.05) appeared in the allele frequencies of rs762551 (*CYP1A2*) and rs156697 (*GSTO2*). However, after Bonferroni correction (threshold for significance = 0.05/13, that is, 0.0038), none of the polymorphisms were significantly different in genotype and allele frequencies between the PD cases and controls.

**TABLE 1:** Genotype and allele frequencies of polymorphisms of CYPs, esterases and GSTs in PD patients and controls.

Polymorphism	Genotype (frequency)			*P* ^a^	Allele (frequency)		*P* ^a^	OR (95% CI)
***CYP1A1* (rs12441817)**	**TT**	**TC**	**CC**		**T**	**C**		
Control	138 (0.277)	242 (0.485)	119 (0.238)		518 (0.519)	480 (0.481)		
PD	150 (0.285)	280 (0.531)	97 (0.184)	0.119	580 (0.550)	474 (0.450)	0.148	0.876 (0.732–1.048)
***CYP1A1* (rs1048943)**	**TT**	**TC**	**CC**		**T**	**C**		
Control	288 (0.577)	174 (0.349)	37 (0.074)		750 (0.752)	248 (0.248)		
PD	328 (0.622)	177 (0.336)	22 (0.042)	0.181	833 (0.790)	221 (0.210)	0.068	0.820 (0.662–1.015)
***CYP1A2* (rs762551)**	**CC**	**CA**	**AA**		**C**	**A**		
Control	66 (0.132)	232 (0.465)	201 (0.403)		364 (0.365)	634 (0.635)		
PD	77 (0.146)	277 (0.526)	173 (0.328)	0.080	431 (0.409)	623 (0.591)	0.043	0.827 (0.688–0.994)
***CYP2C19* (rs4244285)**	**GG**	**GA**	**AA**		**G**	**A**		
Control	213 (0.427)	226 (0.453)	60 (0.120)		652 (0.653)	346 (0.347)		
PD	223 (0.423)	246 (0.467)	58 (0.110)	0.908	692 (0.657)	362 (0.343)	0.839	0.981 (0.812–1.184)
***CYP2E1* (rs2070676)**	**GG**	**GC**	**CC**		**G**	**C**		
Control	12 (0.024)	157 (0.315)	330 (0.661)		181 (0.181)	817 (0.819)		
PD	9 (0.017)	152 (0.288)	366 (0.694)	0.486	170 (0.161)	884 (0.839)	0.266	1.144 (0.903–1.450)
***PON1* (rs662)**	**TT**	**TC**	**CC**		**T**	**C**		
Control	60 (0.120)	235 (0.471)	204 (0.409)		355 (0.356)	643 (0.644)		
PD	75 (0.142)	225 (0.427)	227 (0.431)	0.404	375 (0.356)	679 (0.644)	0.943	1.007 (0.836–1.213)
***PON2* (rs12026)**	**GG**	**GC**	**CC**		**G**	**C**		
Control	329 (0.659)	153 (0.307)	17 (0.034)		811 (0.813)	187 (0.187)		
PD	347 (0.658)	170 (0.323)	10 (0.019)	0.227	864 (0.820)	190 (0.180)	0.712	0.957 (0.760–1.206)
***BCHE* (rs1803274)**	**CC**	**CT**	**TT**		**C**	**T**		
Control	393 (0.788)	101 (0.202)	5 (0.010)		887 (0.889)	111 (0.111)		
PD	417 (0.791)	105 (0.199)	5 (0.009)	0.949	939 (0.891)	115 (0.109)	0.751	0.955 (0.717–1.271)
***GSTM1***	**Present**	**Null**						
Control	279 (0.559)	220 (0.441)						
PD	270 (0.512)	257 (0.488)		0.371				1.123 (0.87–1.448)
***GSTT1***	**Present**	**Null**						
Control	258 (0.517)	241 (0.483)						
PD	295 (0.560)	232 (0.440)		0.252				0.862 (0.669–1.111)
***GSTO1* (rs4925)**	**CC**	**CA**	**AA**		**C**	**A**		
Control	343 (0.687)	141 (0.283)	15 (0.030)		827 (0.829)	171 (0.171)		
PD	380 (0.721)	132 (0.250)	15 (0.028)	0.323	892 (0.846)	162 (0.154)	0.135	0.831 (0.653–1.059)
***GSTO2* (rs156697)**	**AA**	**AG**	**GG**		**A**	**G**		
Control	263 (0.527)	192 (0.385)	44 (0.088)		718 (0.719)	280 (0.281)		
PD	305 (0.579)	189 (0.359)	33 (0.063)	0.105	799 (0.758)	255 (0.242)	0.030	0.799 (0.651–0.979)
***GSTP1* (rs1695)**	**AA**	**AG**	**GG**		**A**	**G**		
Control	320 (0.641)	159 (0.319)	20 (0.040)		799 (0.801)	199 (0.199)		
PD	333 (0.632)	174 (0.330)	20 (0.038)	0.886	840 (0.797)	214 (0.203)	0.631	1.056 (0.845–1.320)

*^a^Adjusted with age and sex.*

*CI, confidence interval; CYPs, cytochromes P450; GSTs, glutathione S-transferases; OR, odds ratio; PD, Parkinson’s disease.*

### Association Analyses of the Polymorphisms With Parkinson’s Disease in Genetic Models

Except for the deletion polymorphisms of *GSTM1* and *GSTT1*, we analyzed the remaining 11 polymorphisms in association with PD using multiple genetic models including dominant, recessive and additive. As shown in Table 2, an association at *P* < 0.05 was present in rs12441817 (*CYP1A1*) and rs762551 (*CYP1A2*) when using the recessive model, and in rs762551 (*CYP1A2*) and rs156697 (*GSTO2*) when using the additive model. However, the associations were no longer considered valid following Bonferroni correction (threshold for significance = 0.05/11, that is, 0.0045).

**TABLE 2:** Association analyses with PD using dominant, recessive, and additive models.

Polymorphism	Genotype	*P* ^a^	OR (95% CI)
***CYP1A1* (rs12441817)**			
Dominant	TT vs. TC + CC	0.68	0.94 (0.71–1.25)
Recessive	TT + TC vs. CC	0.04	0.72 (0.53–0.99)
Additive	TT vs. TC vs. CC	0.14	0.87 (0.73–1.05)
***CYP1A1* (rs1048943)**			
Dominant	TT vs. TC + CC	0.13	0.82 (0.63–1.06)
Recessive	TT + TC vs. CC	0.14	0.66 (0.38–1.15)
Additive	TT vs. TC vs. CC	0.071	0.82 (0.67–1.02)
***CYP1A2* (rs762551)**			
Dominant	CC vs. CA + AA	0.36	0.84 (0.58–1.22)
Recessive	CC + CA vs. AA	0.025	0.74 (0.57–0.96)
Additive	CC vs. CA vs. AA	0.038	0.82 (0.68–0.99)
***CYP2C19* (rs4244285)**			
Dominant	GG vs. GA + AA	0.72	1.05 (0.81–1.35)
Recessive	GG + GA vs. AA	0.9	0.97 (0.65–1.45)
Additive	GG vs. GA vs. AA	0.84	1.02 (0.84–1.23)
***CYP2E1* (rs2070676)**			
Dominant	GG vs. GC + CC	0.4	1.47 (0.60–3.70)
Recessive	GG + GC vs. CC	0.31	1.15 (0.88–1.52)
Additive	GG vs. GC vs. CC	0.25	1.15 (0.90–1.47)
***PON1* (rs662)**			
Dominant	TT vs. TC + CC	0.39	0.85 (0.58–1.23)
Recessive	TT + TC vs. CC	0.49	1.10 (0.85–1.41)
Additive	TT vs. TC vs. CC	0.94	1.01 (0.84–1.20)
***PON2* (rs12026)**			
Dominant	GG vs. GC + CC	0.9	1.02 (0.78–1.33)
Recessive	GG + GC vs. CC	0.094	0.50 (0.22–1.14)
Additive	GG vs. GC vs. CC	0.7	0.96 (0.75–1.21)
***BCHE* (rs1803274)**			
Dominant	CC vs. CT + TT	0.76	0.95 (0.70–1.30)
Recessive	CC + CT vs. TT	0.87	0.90 (0.25–3.20)
Additive	CC vs. CT vs. TT	0.75	0.95 (0.71–1.27)
***GSTO1* (rs4925)**			
Dominant	CC vs. CA + AA	0.14	0.81 (0.61–1.07)
Recessive	CC + CA vs. AA	0.56	0.80 (0.38–1.69)
Additive	CC vs. CA vs. AA	0.14	0.83 (0.66–1.06)
***GSTO2* (rs156697)**			
Dominant	AA vs. AG + GG	0.049	0.77 (0.60–1.00)
Recessive	AA + AG vs. GG	0.16	0.71 (0.44–1.15)
Additive	AA vs. AG vs. GG	0.033	0.80 (0.66–0.98)
***GSTP1* (rs1695)**			
Dominant	AA vs. AG + GG	0.67	1.06 (0.81–1.38)
Recessive	AA + AG vs. GG	0.72	1.13 (0.59–2.16)
Additive	AA vs. AG vs. GG	0.63	1.06 (0.84–1.32)

*^a^Adjusted with age and sex.*

*CI, confidence interval; OR, odds ratio; PD, Parkinson’s disease.*

### Analyses of the Synergistic Effect of the Polymorphisms on Parkinson’s Disease Risk

We divided the 13 polymorphisms into three enzyme classes, GSTs, esterases and CYPs, and assessed the potential synergistic effect of each class on PD risk. To serve this purpose, the genotypes of each SNP were sorted into two groups with the following rationales: the homozygous genotype with frequency lower than 0.05 was clustered into the heterozygous genotype as indicated in “Materials and Methods” section, otherwise the dominant or recessive model with smaller *P*-value was adopted for sorting. The genotypes of *GSTM1* and *GSTT1* were as it is. Group details were listed in the table footnotes. Results showed that none of the genotype combinations were significantly associated with PD within the class of five GSTs, three esterases or five CYPs (threshold for significance at 0.0038 after Bonferroni correction; Supplementary Table 2).

The backward elimination model was then used to detect stronger synergistic effect of the enzymes on PD starting from the combinations with the smallest *P*-value in each class (Supplementary Table 2 and Table 3). Results for CYPs showed that individuals simultaneously carrying the rs12441817/CC (*CYP1A1*), rs762551/AA (*CYP1A2*), and rs2070676/GG + GC genotypes (*CYP2E1*) were more resistant to PD susceptibility (*P* = 0.002, OR = 0.354, 95% CI = 0.184–0.682), while simultaneous presence of the rs12441817/CC and rs2070676/GG + GC genotypes appeared to play the core protective effect (*P* = 0.002, OR = 0.393, 95% CI = 0.216–0.715; Table 3). No significant difference was observed in the gender subgroups. Results for GSTs showed that individuals carrying the *GSTM1*-present, *GSTT1*-null, rs156697/AG + GG *GSTO2*), and rs1695/AA (*GSTP1*) genotypes displayed an increased resistance to PD (*P* = 0.003, OR = 0.348, 95% CI = 0.171–0.706), wherein a gender-dependent effect was observed with significance in females (*P* = 0.003) but not in males (Table 3). Results for esterases suggested no synergistic effect on PD in the total cohort, neither in the gender subgroups (Table 3).

**TABLE 3:** Interaction analyses of polymorphisms of CYPs, esterases and GSTs using backward elimination model.

Genotype^a^	Control, (F)	PD, (F)	*P* ^b^	OR (95% CI)
**CYPs: rs12441817/rs1048943/rs762551/rs4244285/rs2070676**				
**2/2/2/1/1**	14 (0.028)	4 (0.008)	0.020	0.250 (0.077–0.806)
Female	4 (0.017)	1 (0.004)	0.181	0.215 (0.023–2.042)
Male	10 (0.037)	3 (0.011)	0.081	0.289 (0.071–1.168)
**2/2/2/−/1**	27 (0.054)	12 (0.023)	0.008	0.371 (0.177–0.776)
Female	12 (0.052)	5 (0.019)	0.044	0.320 (0.106–0.969)
Male	15 (0.056)	7 (0.026)	0.090	0.423 (0.156–1.145)
**2/−/2/−/1**	34 (0.068)	15 (0.028)	0.002*	0.354 (0.184–0.682)
Female	16 (0.070)	7 (0.027)	0.023	0.334 (0.130–0.857)
Male	18 (0.067)	8 (0.030)	0.028	0.360 (0.145–0.898)
**2/−/−/−/1**	40 (0.080)	18 (0.034)	0.002*	0.393 (0.216–0.715)
Female	17 (0.074)	8 (0.031)	0.050	0.411 (0.169–1.002)
Male	23 (0.085)	10 (0.037)	0.016	0.367 (0.163–0.828)
**Esterases: rs662/rs12026/rs1803274**				
**1/1/1**	21 (0.042)	28 (0.053)	0.402	1.299 (0.704–2.397)
Female	8 (0.035)	16 (0.062)	0.168	1.889 (0.764–4.666)
Male	13 (0.048)	12 (0.044)	0.892	0.943 (0.400–2.223)
**1/1/−**	28 (0.056)	36 (0.068)	0.390	1.265 (0.740–2.164)
Female	11 (0.048)	21 (0.082)	0.167	1.733 (0.794–3.782)
Male	17 (0.063)	15 (0.056)	0.933	0.968 (0.453–2.069)
**GSTs: GSTM1/GSTT1/rs4925/rs156697/rs1695**				
**P/N/1/2/1**	17 (0.034)	5 (0.009)	0.005	0.209 (0.070–0.624)
Female	9 (0.039)	3 (0.012)	0.019	0.178 (0.042–0.752)
Male	8 (0.030)	2 (0.007)	0.098	0.236 (0.043–1.305)
**P/N/−/2/1**	34 (0.068)	17 (0.032)	0.003*	0.348 (0.171–0.706)
Female	22 (0.096)	9 (0.035)	0.003*	0.226 (0.086–0.595)
Male	12 (0.044)	8 (0.030)	0.329	0.584 (0.198–1.719)
**P/N/−/2/−**	57 (0.114)	32 (0.061)	0.013	0.504 (0.293–0.868)
Female	31 (0.135)	15 (0.058)	0.007	0.339 (0.154–0.745)
Male	26 (0.096)	17 (0.063)	0.335	0.688 (0.322–1.472)
**−/N/−/2/−**	117 (0.234)	89 (0.169)	0.018	0.642 (0.444–0.928)
Female	58 (0.253)	44 (0.171)	0.011	0.498 (0.291–0.850)
Male	59 (0.219)	45 (0.167)	0.321	0.771 (0.462–1.288)

*^a^“1” and “2”, respectively, denote TT + TC and CC for rs12441817, TT and TC + CC for rs1048943, CC + CA and AA for rs762551, GG and GA + AA for rs4244285, GG + GC and CC for rs2070676, TT and TC + CC for rs662, GG and GC + CC for rs12026, CC and CT + TT for rs1803274, CC and CA + AA for rs4925, AA and AG + GG for rs156697, and AA and AG + GG for rs1695, respectively. “P” and “N” respectively denote present and null for GSTM1 and GSTT1 variants. The grouping rationale was described in “Results” section.*

*^b^Adjusted with age and sex.*

*P < 0.0038. CI, confidence interval; CYPs, cytochromes P450; F, frequency; GSTs, glutathione S-transferases; OR, odds ratio; PD, Parkinson’s disease.

## Discussion

Xenobiotic metabolizing enzymes are key to the retention and deposition of environmental substances in human bodies. The exposures, in particular pesticide, have been well-recognized to aggravate PD risk. The association between pesticide and PD may be more prominent in eastern China due to the higher pesticide residues (Yu et al., 2020). By analyzing 13 functional or potential PD-associated polymorphisms in genes which encode three classes of metabolizing enzymes, including GSTs, CYPs, and esterases, we demonstrate that none of the polymorphisms are independently associated with PD risk. These observations are in line with results of a previous genome-wide association study (GWAS) in East Asian (Foo et al., 2020). On the other hand, there are synergistic effects on PD in polymorphisms of *CYPs* and *GSTs*, that is, the protection conferred, respectively, by simultaneous presence of the rs12441817/CC (*CYP1A1*) and rs2070676/GG + GC (*CYP2E1*) genotypes, as well as of the *GSTM1*-present, *GSTT1*-null, rs156697/AG + GG (*GSTO2*) and rs1695/AA (*GSTP1*) genotypes.

CYPs are the class of the most important phase I enzymes responsible for metabolism of various xenobiotic compounds, including drugs and pesticides (Elfaki et al., 2018). Genetic polymorphisms in *CYPs* are associated with altered enzymatic activities and disturbed expression levels. The changes in activity or expression may lead to inter-individual variations in xenobiotic metabolisms, and are implicated in various disease susceptibilities (Dardiotis et al., 2013; Elfaki et al., 2018). In context of PD, the C allele of rs12441817 (*CYP1A1*) was shown to be associated with reduced risk of PD (*P* = 5.22 × 10^–5^, OR = 0.87, 95% CI = 0.81–0.93) in a European-descent based GWAS (Nalls et al., 2014). However, this association is not confirmed by the current study and the Asian GWAS (Foo et al., 2020). We are not sure whether it is ethnicity specific. In addition, rs2070676 (*CYP2E1*) showed an association with PD in a Swedish population (Shahabi et al., 2009), rs1048943 (*CYP1A1*) was associated with PD in a Japanese population (Takakubo et al., 1996), and rs762551 (*CYP1A2*) was reported to have a marginal association with PD risk in the United States and Denmark (Palacios et al., 2010; Chuang et al., 2016). Nonetheless, these associations are not independently observed in the current study, the Asian GWAS (Foo et al., 2020), the European GWAS (Nalls et al., 2014), and other case-control studies (Chan et al., 2002; Facheris et al., 2008; Siokas et al., 2021). As noted in Supplementary Table 1, the rs1048943 is a missense variant of *CYP1A1* (T > C, Ile462Val) and functionally regulates the CYP1A1 enzyme activity (Shah et al., 2009). The CYP1A2 activity is also influenced by its rs762551 polymorphism (Denden et al., 2016). The rs4244285 is the most studied polymorphism of *CYP2C19*, and can alter the *CYP2C19* mRNA reading frame leading to a truncated non-functional protein (Scott et al., 2012). As discussed above, these polymorphisms are not, at least not consistently, shown to be associated with PD, suggesting that bluntness of each enzyme *per se* may not be sufficient to affect PD susceptibility. On the other hand, our results demonstrate that simultaneous presence of the rs12441817/CC (*CYP1A1*) and rs2070676/GG + GC (*CYP2E1*) genotypes confers protection against PD risk, suggesting a synergistic genetic effect from these two enzymes. Coincidentally, the rs12441817/C and the rs2070676/G indeed served as the protective allele in the two positive studies (Shahabi et al., 2009; Nalls et al., 2014), which to some extent supports our finding. These results together indicate that a synergistic play more likely leads to a modulation of PD risk than individually.

Esterases, including paraoxonase 1 (PON1), paraoxonase 2 (PON2), and butyrylcholinesterase (BCHE), are responsible for catalyzing ester hydrolysis during Phase I reactions, and are critical in organophosphate metabolism (Satoh et al., 2002). Two of the most studied polymorphisms of *PON1* are rs854560 (A > T, Leu55Met) and rs662 (T > C, Gln192Arg). The rs854560/A and rs662/C alleles result in higher PON1 enzyme activity (Rejeb et al., 2013). The rs854560 was excluded from the current study due to a low minor allele frequency in East Asians (T allele at 0.034; from gnomAD).^1^ The rs12026 of *PON2* (G > C, Ala148Gly) and rs1803274 of *BCHE* (G > A, Ala539Thr) are linked with lower enzyme activity at presence of the C and A allele, respectively (Rejeb et al., 2013; Habieb et al., 2021). Nonetheless, neither individual nor synergistic effect on PD risk is detected in these variants, suggesting that esterases may not be important in modulating the environmental and genetic interplay toward PD risk. Besides, there are two previous reports for rs662 and PD with results showing positive association (Kondo and Yamamoto, 1998) and no association (Wang and Liu, 2000).

The current study detects no individual association between the *GST* polymorphisms and PD risk. The rs4925 (*GSTO1*), rs156697 (*GSTO2*), and rs1695 (*GSTP1*) have not been reported in Chinese PD cohorts till now. The *GSTM1* and *GSTT1* deletion polymorphisms have been previously reported in relatively small Chinese populations, also showing no association with PD (Tao et al., 1998; Fong et al., 2006). The lack of PD association in these variants has been additionally observed in other ethnicities, including Caucasians, Australians and Japanese (Menegon et al., 1998; Ahmadi et al., 2000; Harada et al., 2001; Whitbread et al., 2004). Nonetheless, reports of significant association with PD also exist, such as for the *GSTM1* variant in Chileans (Perez-Pastene et al., 2007), the *GSTT1* variant in a UK population (De Palma et al., 1998), and the rs4925 in Americans (Wahner et al., 2007). Regardless of the individual performance, the present study for the first time reports a protection against PD synergistically conferred by the genotypes of *GSTM1*-present, *GSTT1*-null, rs156697/AG + GG (*GSTO2*) and rs1695/AA (*GSTP1*). The *GSTM1*-present and the rs1695/A allele are linked with elevated enzyme activities (Johansson et al., 1998; Hayes and Strange, 2000). In contrast, the *GSTT1*-null and the rs156697/G lead to reduced enzyme activities or protein levels (Hayes and Strange, 2000; Allen et al., 2012). Thus, the combination of these four genotypes appears to be mediocre in terms of collective enzyme activity. Interestingly, there is a similar case showing a synergistic effect from the alleles of *GSTM1*-null, *GSTT1*-present, *GSTA1*/T (low activity), and *GSTP1*/G (low activity) on promoting risk for clear cell renal cell carcinoma (Coric et al., 2016). In certain cases, high GST activity may lead to increased toxicity, such that GSTT1 dehalogenates and transforms halogenated organic compounds to toxic products (Landi, 2000). In addition, the expression of *GSTs* may be regulated by sex hormones (Coecke et al., 2000), which may partially explain our observed female-specific synergistic effect.

## Conclusion

In conclusion, the current study demonstrates that polymorphisms of *CYPs* and *GSTs*, but not those of esterases, have synergistic impact on PD susceptibility. The variants are not associated with PD individually. Our findings suggest that more attention should be paid to the synergistic interplay for the genetic variations of xenobiotic metabolizing enzymes to better understand their roles in PD risk.

## Data Availability Statement

The datasets presented in this study can be found in online repositories. The names of the repository/repositories and accession number(s) can be found below: https://datadryad.org/stash, https://datadryad.org/stash/share/bcA9u9G-dGMdGLw_TnpEtY9qPeJg7aXdRA7u35_tgxs.

## Ethics Statement

The studies involving human participants were reviewed and approved by the Ethics Committee of the Second Affiliated Hospital and Yuying Children’s hospital, Wenzhou Medical University. The patients/participants provided their written informed consent to participate in this study.

## Author Contributions

H-HF designed the study. H-HF, K-YW, H-DY, M-JG, X-HY, and H-JD conducted the experiments. H-HF and J-HZ analyzed the data. B-QL and XZ contributed samples. H-HF and J-HZ wrote the manuscript. XZ and J-HZ supervised the study. All authors contributed to the article and approved the submitted version.

## Conflict of Interest

The authors declare that the research was conducted in the absence of any commercial or financial relationships that could be construed as a potential conflict of interest.

## Publisher’s Note

All claims expressed in this article are solely those of the authors and do not necessarily represent those of their affiliated organizations, or those of the publisher, the editors and the reviewers. Any product that may be evaluated in this article, or claim that may be made by its manufacturer, is not guaranteed or endorsed by the publisher.

## Footnotes

1
https://gnomad.broadinstitute.org/
