# Supplementary material for: Polymorphisms of Cytochromes P450 and Glutathione S-Transferases Synergistically Modulate Risk for Parkinson’s Disease
Source: Front Aging Neurosci. 2022 Apr 29;14:888942. doi: 10.3389/fnagi.2022.888942 (PMC9099289; doi:10.3389/fnagi.2022.888942)
Supplement: Supplementary file 1 [file Data_Sheet_1.docx]

**Table S1. Summary of the candidate polymorphisms**

| **Gene** | **Polymorphism, allele Ref > Alt** | **Function^a^** | **Reference** |
| --- | --- | --- | --- |
| ***CYPs*** |  |  |  |
| *CYP1A1* | rs12441817, T > C | May reduce PD risk | Nalls et al., 2014 |
| *CYP1A1* | rs1048943, T > C | Missense, increase activity | Shah et al., 2009 |
| *CYP1A2* | rs762551, C > A | Influence activity | Denden et al., 2016 |
| *CYP2C19* | rs4244285, G > A | Reduce activity | Scott et al., 2012 |
| *CYP2E1* | rs2070676, G > C | Increase PD risk | Shahabi et al., 2009 |
| ***Esterases*** |  |  |  |
| *PON1* | rs662, T > C | Missense, increase activity | Rejeb et al., 2013 |
| *PON2* | rs12026, G > C | Missense, reduce activity | Rejeb et al., 2013 |
| *BCHE* | rs1803274, C > T | Missense, reduce activity | Habieb et al., 2021 |
| ***GSTs*** |  |  |  |
| *GSTM1* | deletion, Present > Null | Reduce activity | Hayes et al., 2000 |
| *GSTT1* | deletion, Present > Null | Reduce activity | Hayes et al., 2000 |
| *GSTO1* | rs4925, C > A | Missense, reduce activity | Tanaka-Kagawa et al., 2003 |
| *GSTO2* | rs156697, A > G | Missense, decrease expression | Allen et al., 2012 |
| *GSTP1* | rs1695, A > G | Missense, reduce activity | Johansson et al., 1998 |

^a^ Consequence of the Ref allele changed to the Alt allele.

Alt, alternate; CYPs, cytochromes P450; GSTs, glutathione S-transferases; PD, Parkinson’s disease; Ref, reference.

**Table S2. Interaction analyses of polymorphisms of CYPs, esterases and GSTs**

| **Genotype ^a^** | **Control, n (%)** | **PD, n (%)** | ***P* ^b^** | **OR (95% CI)** |
| --- | --- | --- | --- | --- |
| **CYPs: rs12441817/rs1048943/rs762551/rs4244285/rs2070676** | | | | |
| 1/1/1/2/2 | 85 (17) | 93 (17.6) | reference | reference |
| 1/1/1/1/1 | 31 (6.2) | 24 (4.6) | 0.267 | 0.702 (0.376-1.311) |
| 1/2/1/1/1 | 10 (2) | 14 (2.7) | 0.633 | 1.239 (0.514-2.988) |
| 2/2/1/1/1 | 2 (0.4) | 1 (0.2) | 0.359 | 0.312 (0.026-3.751) |
| 1/1/2/1/1 | 11 (2.2) | 11 (2.1) | 0.874 | 1.077 (0.432-2.684) |
| 1/2/2/1/1 | 2 (0.4) | 6 (1.1) | 0.394 | 2.079 (0.387-11.171) |
| 2/1/2/1/1 | 3 (0.6) | 2 (0.4) | 0.511 | 0.532 (0.081-3.494) |
| 2/2/2/1/1 | 14 (2.8) | 4 (0.8) | 0.02 | 0.25 (0.077-0.806) |
| 1/1/1/1/2 | 50 (10) | 70 (13.3) | 0.375 | 1.244 (0.768-2.015) |
| 1/2/1/1/2 | 23 (4.6) | 28 (5.3) | 0.799 | 1.087 (0.572-2.067) |
| 2/1/1/1/2 | 2 (0.4) | 5 (0.9) | 0.470 | 1.939 (0.322-11.69) |
| 2/2/1/1/2 | 8 (1.6) | 2 (0.4) | 0.161 | 0.316 (0.063-1.584) |
| 1/1/2/1/2 | 21 (4.2) | 13 (2.5) | 0.042 | 0.451 (0.209-0.973) |
| 1/2/2/1/2 | 12 (2.4) | 9 (1.7) | 0.409 | 0.672 (0.262-1.726) |
| 2/1/2/1/2 | 5 (1) | 12 (2.3) | 0.268 | 1.875 (0.617-5.701) |
| 2/2/2/1/2 | 19 (3.8) | 22 (4.2) | 0.791 | 1.099 (0.545-2.215) |
| 1/1/1/2/1 | 33 (6.6) | 48 (9.1) | 0.322 | 1.319 (0.763-2.28) |
| 1/2/1/2/1 | 20 (4) | 15 (2.8) | 0.211 | 0.618 (0.29-1.314) |
| 2/1/1/2/1 | 1 (0.2) | 0 (0) | 1 | - |
| 2/2/1/2/1 | 3 (0.6) | 2 (0.4) | 0.457 | 0.49 (0.075-3.205) |
| 1/1/2/2/1 | 13 (2.6) | 18 (3.4) | 0.593 | 1.242 (0.561-2.751) |
| 1/2/2/2/1 | 9 (1.8) | 7 (1.3) | 0.748 | 0.84 (0.29-2.431) |
| 2/1/2/2/1 | 4 (0.8) | 1 (0.2) | 0.275 | 0.29 (0.031-2.675) |
| 2/2/2/2/1 | 13 (2.6) | 8 (1.5) | 0.273 | 0.584 (0.224-1.527) |
| 1/2/1/2/2 | 27 (5.4) | 46 (8.7) | 0.213 | 1.444 (0.81-2.574) |
| 2/1/1/2/2 | 0 (0) | 3 (0.6) | 0.999 | - |
| 2/2/1/2/2 | 3 (0.6) | 3 (0.6) | 0.96 | 0.958 (0.18-5.095) |
| 1/1/2/2/2 | 16 (3.2) | 19 (3.6) | 0.498 | 1.298 (0.611-2.756) |
| 1/2/2/2/2 | 17 (3.4) | 9 (1.7) | 0.11 | 0.486 (0.2-1.179) |
| 2/1/2/2/2 | 13 (2.6) | 9 (1.7) | 0.350 | 0.644 (0.256-1.622) |
| 2/2/2/2/2 | 29 (5.8) | 23 (4.4) | 0.263 | 0.692 (0.364-1.318) |
| **Esterases: rs662/rs12026/rs1803274** | | | | |
| 2/1/1 | 239 (47.9) | 254 (48.2) | reference | reference |
| 1/1/1 | 21 (4.2) | 28 (5.3) | 0.402 | 1.299 (0.704-2.397) |
| 1/1/2 | 7 (1.4) | 8 (1.5) | 0.909 | 1.064 (0.365-3.105) |
| 1/2/1 | 28 (5.6) | 31 (5.9) | 0.947 | 0.981 (0.563-1.711) |
| 1/2/2 | 4 (0.8) | 8 (1.5) | 0.287 | 1.971 (0.565-6.875) |
| 2/1/2 | 62 (12.4) | 57 (10.8) | 0.616 | 0.899 (0.594-1.362) |
| 2/2/1 | 105 (21) | 104 (19.7) | 0.939 | 1.013 (0.725-1.416) |
| 2/2/2 | 33 (6.6) | 37 (7) | 0.881 | 0.961 (0.572-1.615) |
| **GSTs: GSTM1/ GSTT1/rs4925/rs156697/rs1695** | | | | |
| P/P/1/1/1 | 51 (10.2) | 71 (13.5) | reference | reference |
| N/N/1/1/1 | 35 (7) | 36 (6.8) | 0.354 | 0.749 (0.407-1.38) |
| N/N/1/1/2 | 18 (3.6) | 31 (5.9) | 0.895 | 1.049 (0.520-2.117) |
| N/N/1/2/1 | 19 (3.8) | 12 (2.3) | 0.043 | 0.425 (0.185-0.974) |
| N/N/1/2/2 | 4 (0.8) | 9 (1.7) | 0.49 | 1.558 (0.443-5.485) |
| N/N/2/2/1 | 22 (4.4) | 27 (5.1) | 0.604 | 0.833 (0.418-1.661) |
| N/N/2/2/2 | 15 (3) | 9 (1.7) | 0.056 | 0.401 (0.157-1.024) |
| N/P/1/1/1 | 37 (7.4) | 41 (7.8) | 0.268 | 0.716 (0.397-1.293) |
| N/P/1/1/2 | 20 (4) | 30 (5.7) | 0.756 | 1.115 (0.560-2.221) |
| N/P/1/2/1 | 5 (1) | 16 (3) | 0.122 | 2.374 (0.794-7.104) |
| N/P/1/2/2 | 11 (2.2) | 5 (0.9) | 0.014 | 0.23 (0.071-0.74) |
| N/P/2/2/1 | 20 (4) | 23 (4.4) | 0.563 | 0.808 (0.393-1.662) |
| N/P/2/2/2 | 14 (2.8) | 18 (3.4) | 0.498 | 0.757 (0.338-1.693) |
| P/N/1/1/1 | 46 (9.2) | 44 (8.3) | 0.229 | 0.705 (0.400-1.245) |
| P/N/1/1/2 | 25 (5) | 32 (6.1) | 0.927 | 0.97 (0.502-1.871) |
| P/N/1/2/1 | 17 (3.4) | 5 (0.9) | 0.005 | 0.209 (0.07-0.624) |
| P/N/1/2/2 | 5 (1) | 2 (0.4) | 0.156 | 0.278 (0.048-1.626) |
| P/N/2/2/1 | 17 (3.4) | 12 (2.3) | 0.09 | 0.479 (0.205-1.122) |
| P/N/2/2/2 | 18 (3.6) | 13 (2.5) | 0.198 | 0.585 (0.259-1.323) |
| P/P/1/1/2 | 31 (6.2) | 20 (3.8) | 0.059 | 0.514 (0.258-1.024) |
| P/P/1/2/1 | 10 (2) | 16 (3) | 0.908 | 1.054 (0.433-2.566) |
| P/P/1/2/2 | 9 (1.8) | 10 (1.9) | 0.946 | 0.966 (0.354-2.638) |
| P/P/2/2/1 | 41 (8.2) | 30 (5.7) | 0.018 | 0.477 (0.258-0.883) |
| P/P/2/2/2 | 9 (1.8) | 15 (2.8) | 0.833 | 1.107 (0.429-2.857) |

^a^ “1” and “2” respectively denote TT+TC and CC for rs12441817, TT and TC+CC for rs1048943, CC+CA and AA for rs762551, GG and GA+AA for rs4244285, GG+GC and CC for rs2070676, TT and TC + CC for rs662, GG and GC+CC for rs12026, CC and CT+TT for rs1803274, CC and CA+AA for rs4925, AA and AG+GG for rs156697, and AA and AG+GG for rs1695, respectively. “P” and “N” respectively denote present and null for *GSTM1* and *GSTT1* variants. The grouping rationale was described in the Results section.

^b^ Adjusted with age and sex.

CI, confidence interval; CYPs, cytochromes P450; GSTs, glutathione S-transferases; OR, odds ratio; PD, Parkinson’s disease
